# Isolated Terminal Myelocystocele: A Rare Spinal Dysraphism

**Published:** 2011-03-10

**Authors:** Bilal Mirza, Nasir Mahmood, Lubna Ijaz, Tariq Khawaja, Imaran Aslam, Afzal Sheikh

**Affiliations:** Department of Pediatric Surgery, The Children's Hospital and the Institute of Child Health Lahore, Pakistan

**Keywords:** Terminal myelocystocele, Spinal dysraphism, Myelomeningocele

## Abstract

Terminal myelocystocele is a rare spinal dysraphism that present as lumbosacral mass. Magnetic resonance imaging (MRI) is the modality of choice for preoperative diagnosis. A 2.5 months old female baby presented with lumbosacral skin covered mass. There were no associated neurological deficits. MRI of the lesion suggested two cysts, one of which was continuous with the central canal of the spinal cord. At operation terminal myelocystocele was found with tethering of the spinal cord. Untethering of the spinal cord and repair of the myelocystocele performed with uneventful recovery.

## INTRODUCTION

Terminal myelocystocele (TMC) is a rare spinal cord anomaly comprising 4-8% of all cases of spinal dysraphism. Many a time it is associated with other congenital anomalies such as anorectal malformations, abdominal wall defects, spinal anomalies, urogenital anomalies etc. A number of these patients also have associated neurological deficit; however few case reports described no neurological deficits even after surgical repair. Isolated terminal myelocystocele is rarely associated with neurological deficits [1, 2]. This report describes a rare type of spinal dysraphism.

## CASE REPORT

A 2.5-month-old female baby presented with a large lumbosacral skin covered swelling which was present since birth. The patient was a product of consanguineous marriage and born through vaginal delivery. The swelling gradually increased in size. No abnormality was reported in relation to the urination and defecation. Head circumference was 38cm and the neurological examination was essentially normal.

The swelling was smooth, cystic, fluctuant and transluminent, present over lumbosacral region measuring 8X6 cm2 in size with obliteration of natal cleft (Fig. 1). Ultrasound showed a large cystic swelling with a defect in the spine through which meninges were protruding. An MRI depicted a double compartment cystic swelling. The inner cyst was a continuation of the spinal cord central canal that too was dilated (hydromyelia). The cyst was seen protruding from a defect in the posterior osseous elements of the lower lumbar and sacral vertebrae. The spinal cord was tethered and low lying (Fig. 2, 3).

**Figure:**
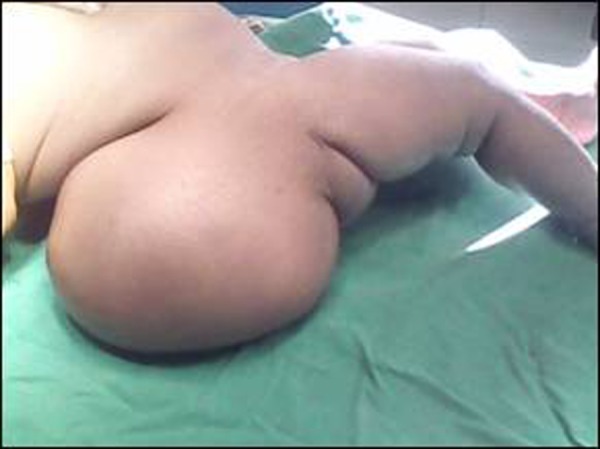
Figure 1: A large lumbo-sacral skin covered mass

**Figure:**
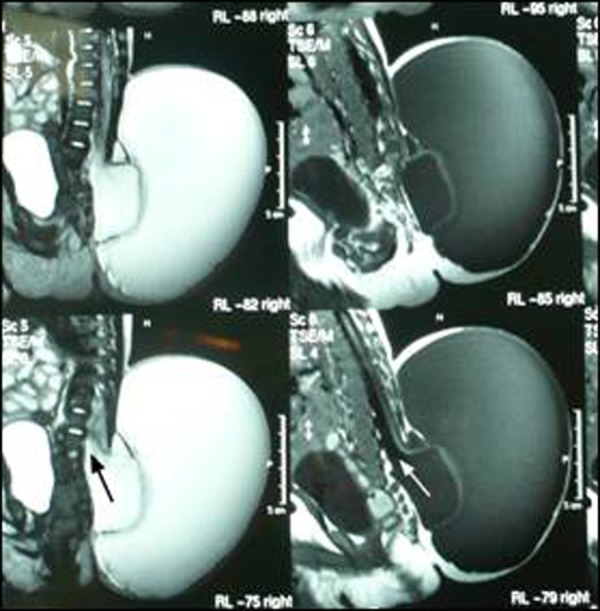
Figure 2: MRI sagittal section showing two cysts. The inner cyst was in continuation (arrow) with the central canal of the spinal cord

**Figure:**
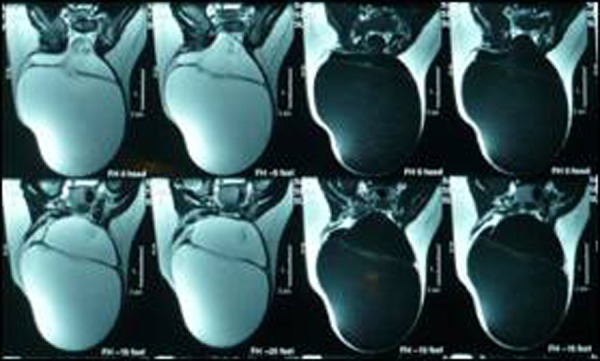
Figure 3: MRI in coronal section showing double compartment swelling

The patient was operated electively. A vertical incision was made in the midline and about 250ml cerebrospinal fluid (CSF) drained. On further exploration of the external cyst, another small cyst was found close to the spine.This cyst was also opened and found to be in continuation with the central canal. The spinal cord ended slightly cephalad with a fibrous band tethering the cord to the dorsal and cephalic aspects of the inner cyst (Fig. 4). The operative diagnosis was terminal myelocystocele. The untethering of the spinal cord was done after which the water-tight repair of the defect performed. The postoperative recovery was uneventful. The patient is on follow up and doing well.

**Figure:**
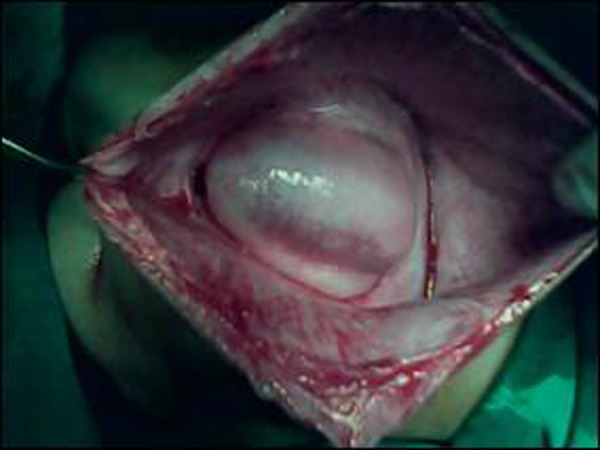
Figure 4: Another cyst inside the major cyst- the terminal myelocystocele

## DISCUSSION

 Neural tube defects (NTD) are perhaps the most frequently occurring congenital anomalies. The incidence is 1:1000 births, but variable throughout the world. They usually occur during 3rd - 4th gestational weeks. Embryologically, a failure of or defective neural tube formation results in NTD. They may occur focally or at multiple points [3].

Spina bifida is a term used to describe the NTD occurring in the spine. It is classified as occulta and cystica/aperta. Spina bifida occulta is a skin covered defect of the vertebral arches without neuronal involvement. Very often found in the lumbo-sacral region and accounts for 10% of otherwise normal individuals. The associated neurological dysfunction is absent or negligible. It can be identified as a tuft of hair, a hemangioma, a sinus etc. at lower back. Spina bifida cystic/aperta is a severe form of NTD and characterized by a defect of vertebral arches through which meninges and the neuronal tissue protrudes into a sac. They often associated with neurological deficits. Meningomyelocele is usually associated with Arnold-chiari malformations and hydrocephalus in more than 90% of cases [3]. Myelomeningocele is the frequently occurring spina bifida whereas terminal myelocystocele accounts for 4-8% of all cases of spinal dysraphism. Antenatal detection of the myelocystocele remained challenging as to its differential diagnosis [3, 4, 5, 6].

MRI, both fetal and after birth, is an important diagnostic tool for the index condition. It can delineate a cystic mass with septation in a coronal view. In sagittal view it can show the continuation between central canal of the spinal cord and the inner cyst. The communication of the outer cyst with subarachnoid space can also be visualized. The other spinal and cord anomalies like tethered cord, diastematomyelia, Arnold-chiari malformations, syringocele etc. can also be detected with this tool. Antenatal ultrasonography in expert hands can delineate spinal dysraphism but it is very difficult to diagnose myelocystocele with accuracy even if performed after birth [4, 5, 6].

Myelocystocele have been reported in cervical, thoracic and lumbosacral regions. Cervical myelocystocele is infrequently associated with neurological deficit whereas terminal myelocystocele is considered to have more neurological problems [6]. Our case was an isolated myelocystocele with no neurological problem.

To summarize, myelocystocele is a rare spinal dysraphism and rarer still is an isolated terminal myelocystocele with very negligible neurological deficit. MRI can diagnose the condition in-utero as well as postnatally. Excellent outcome can be achieved by an early repair of the defect.

## Footnotes

**Source of Support:** Nil

**Conflict of Interest:** None declared
